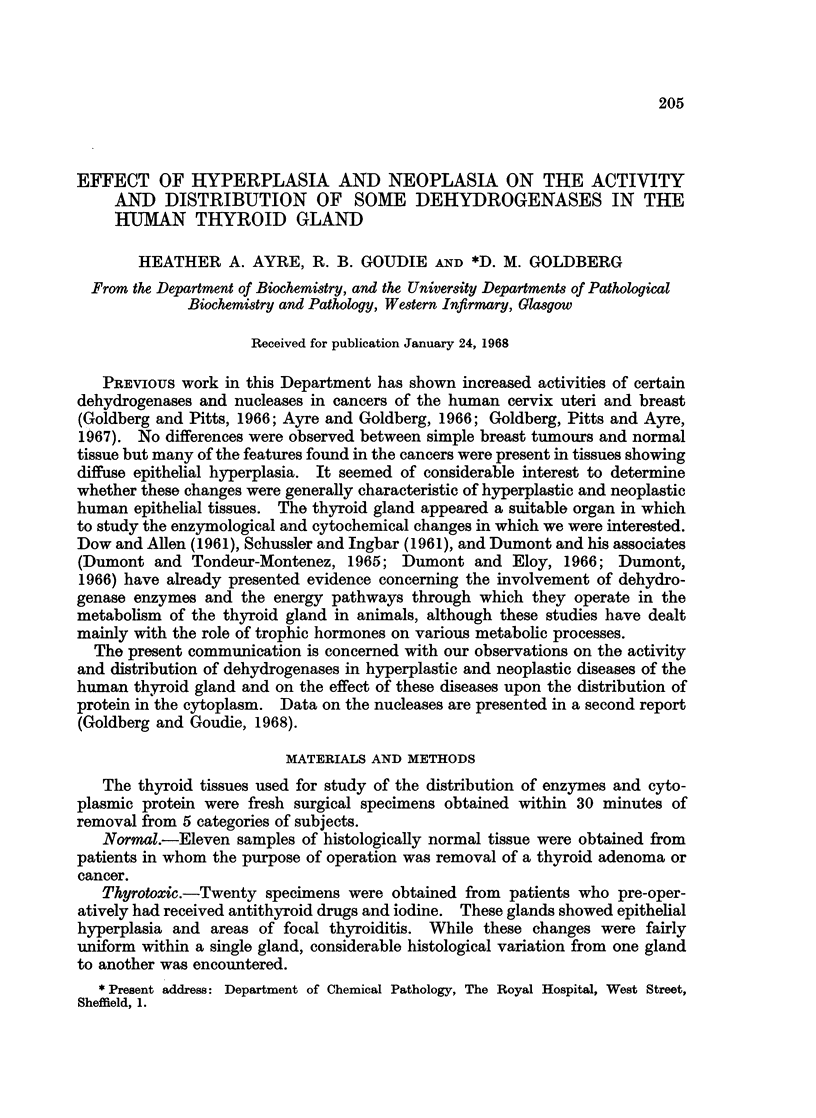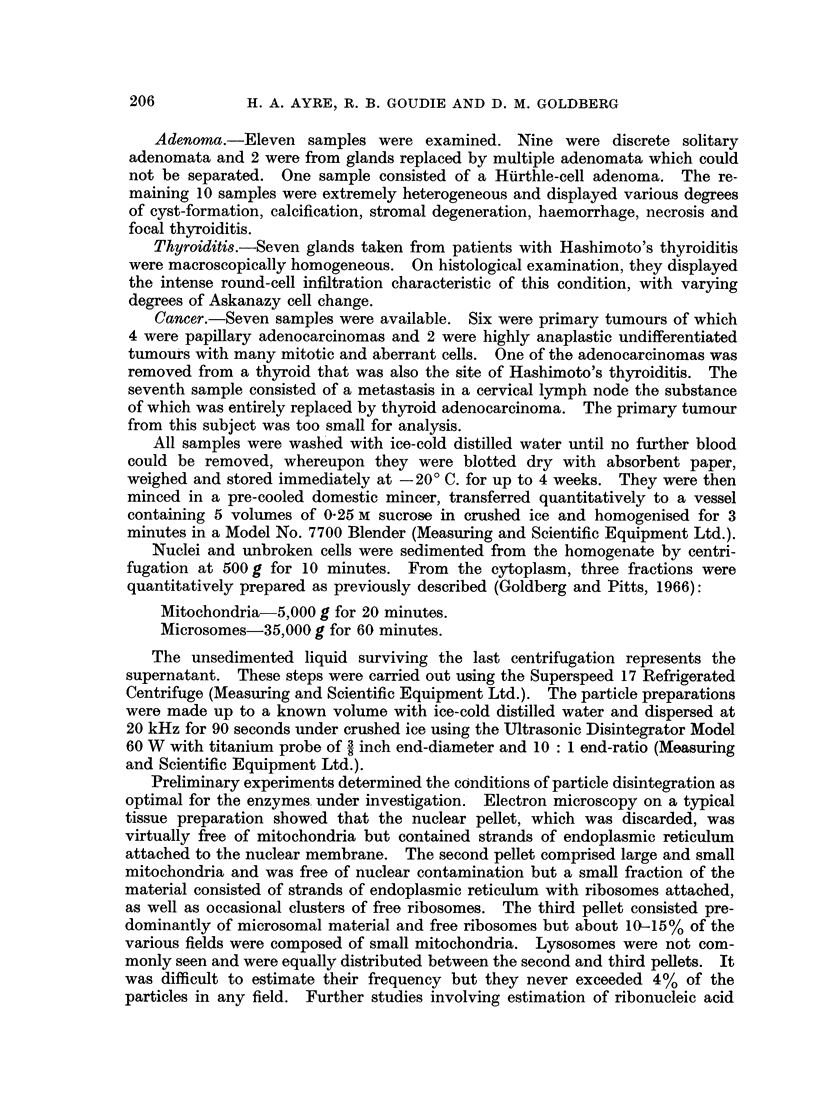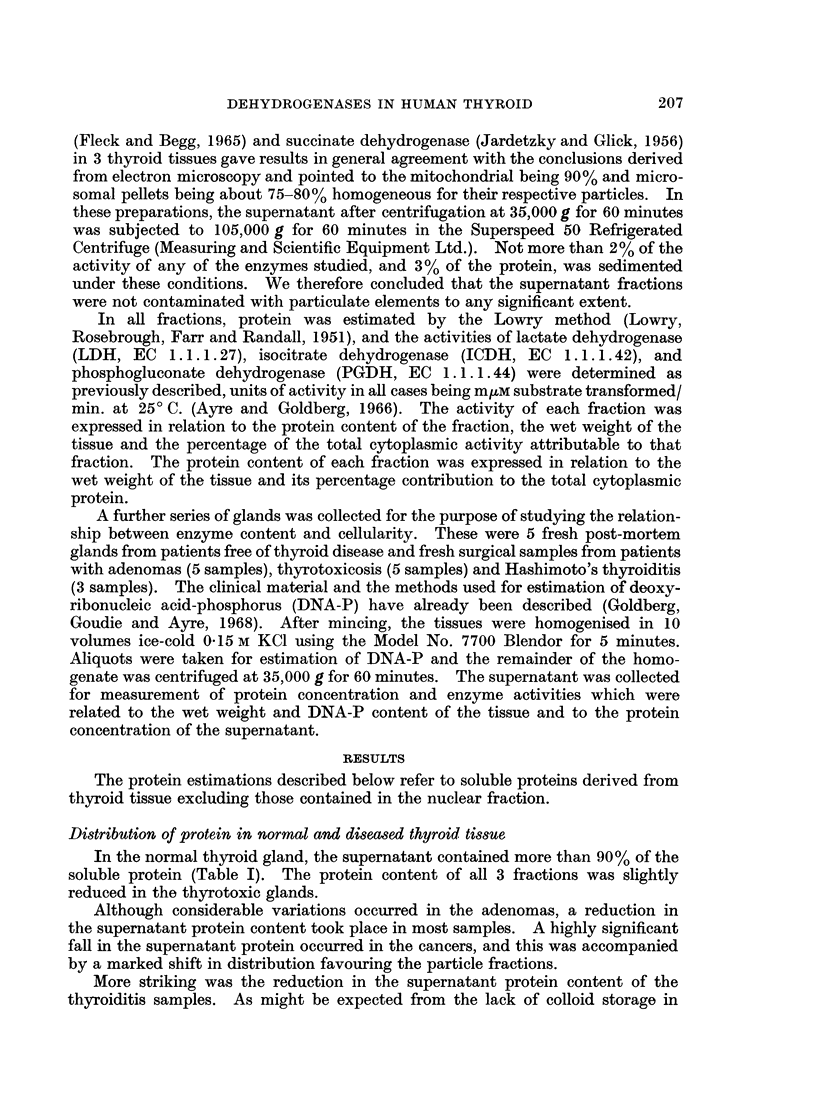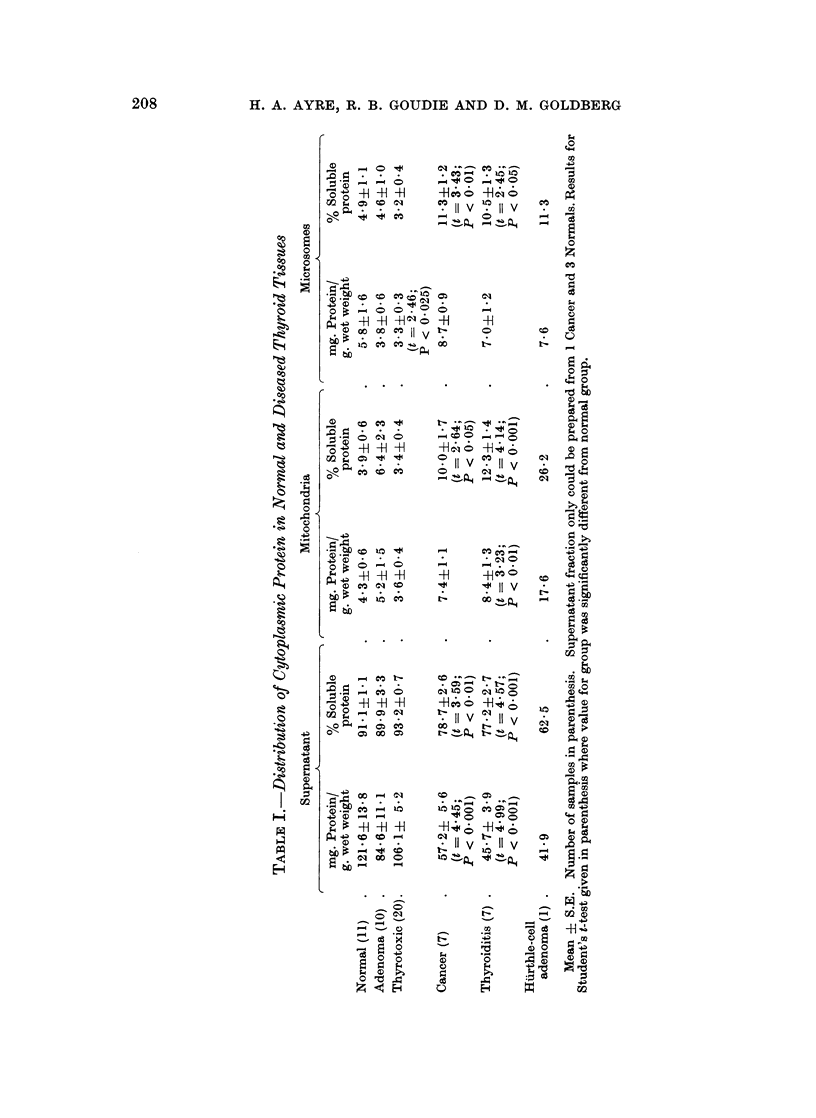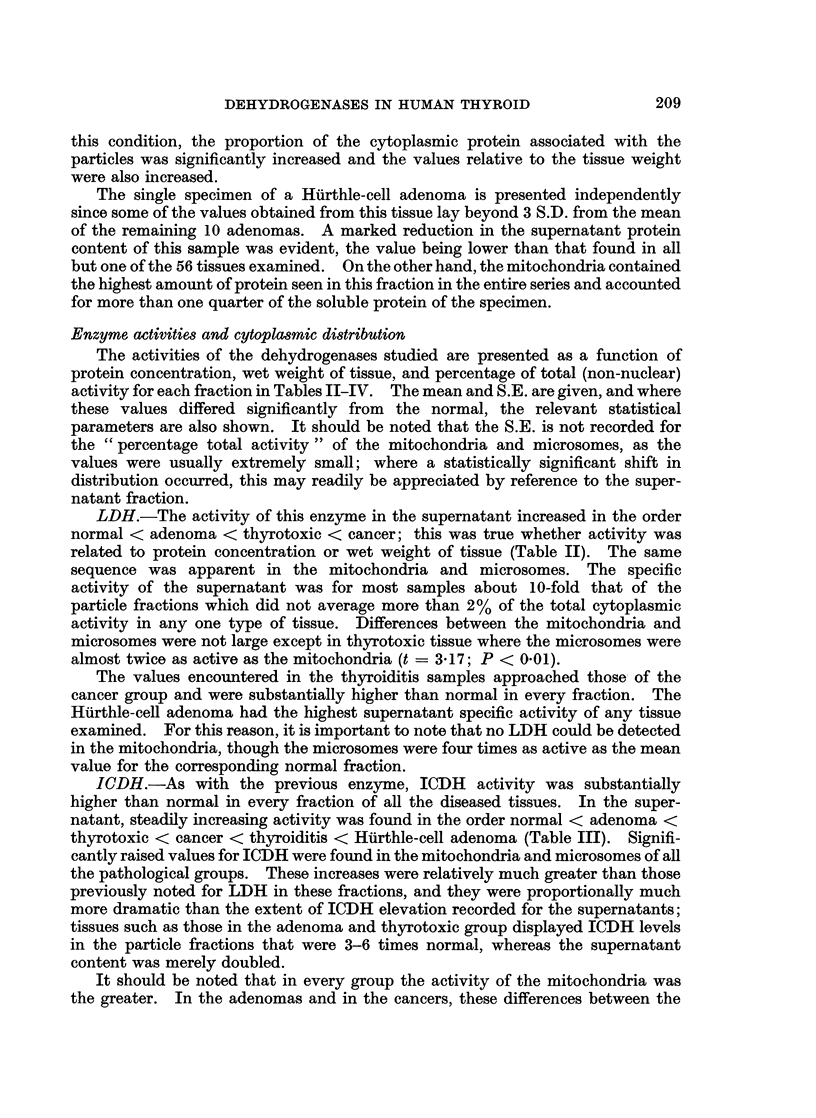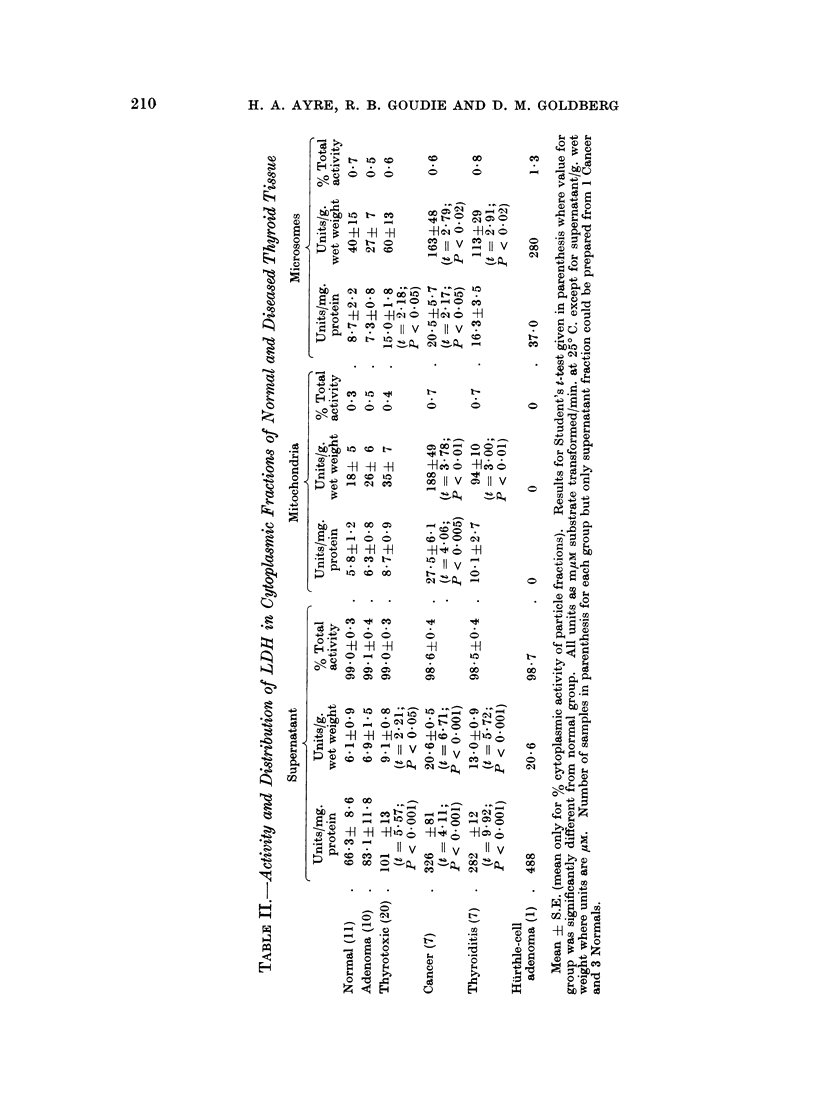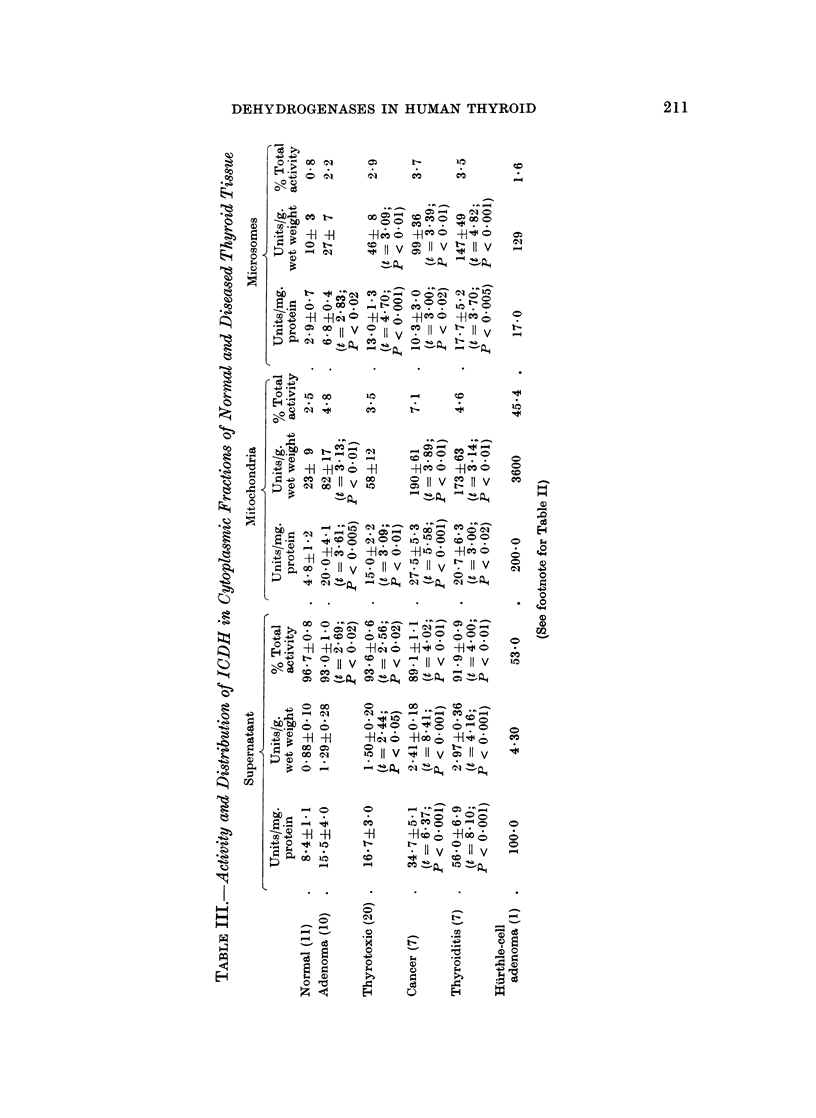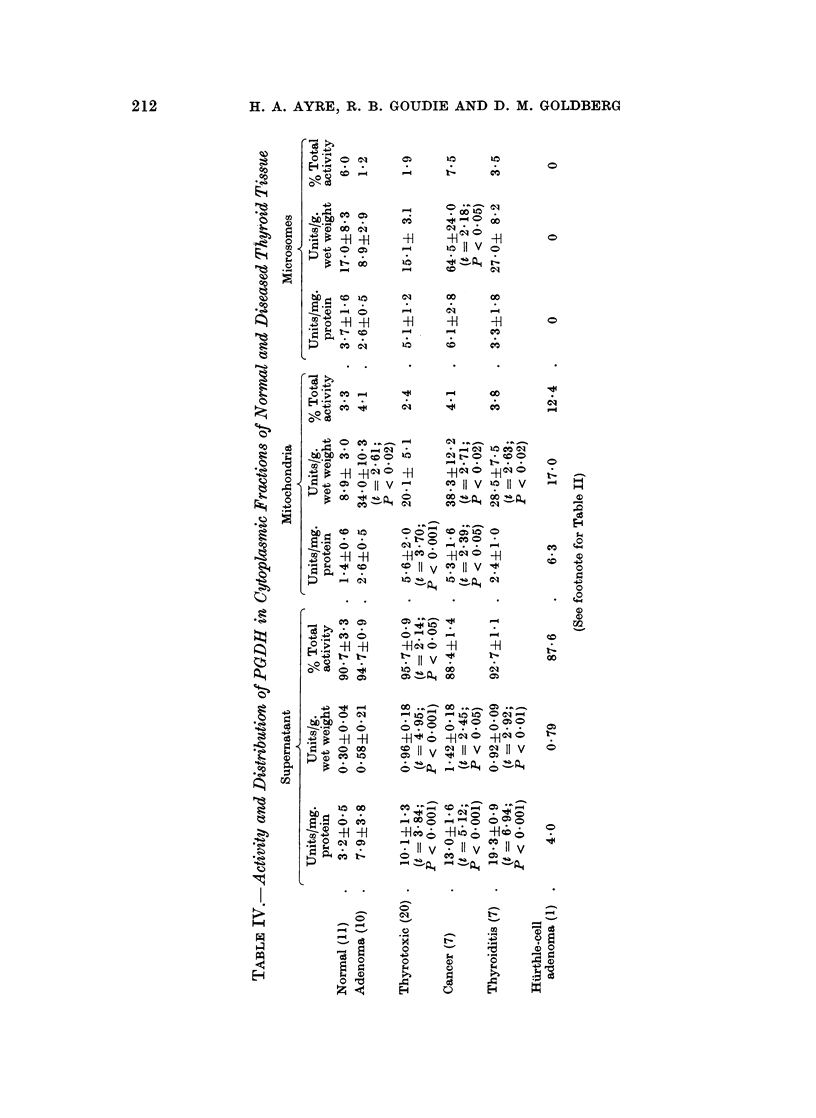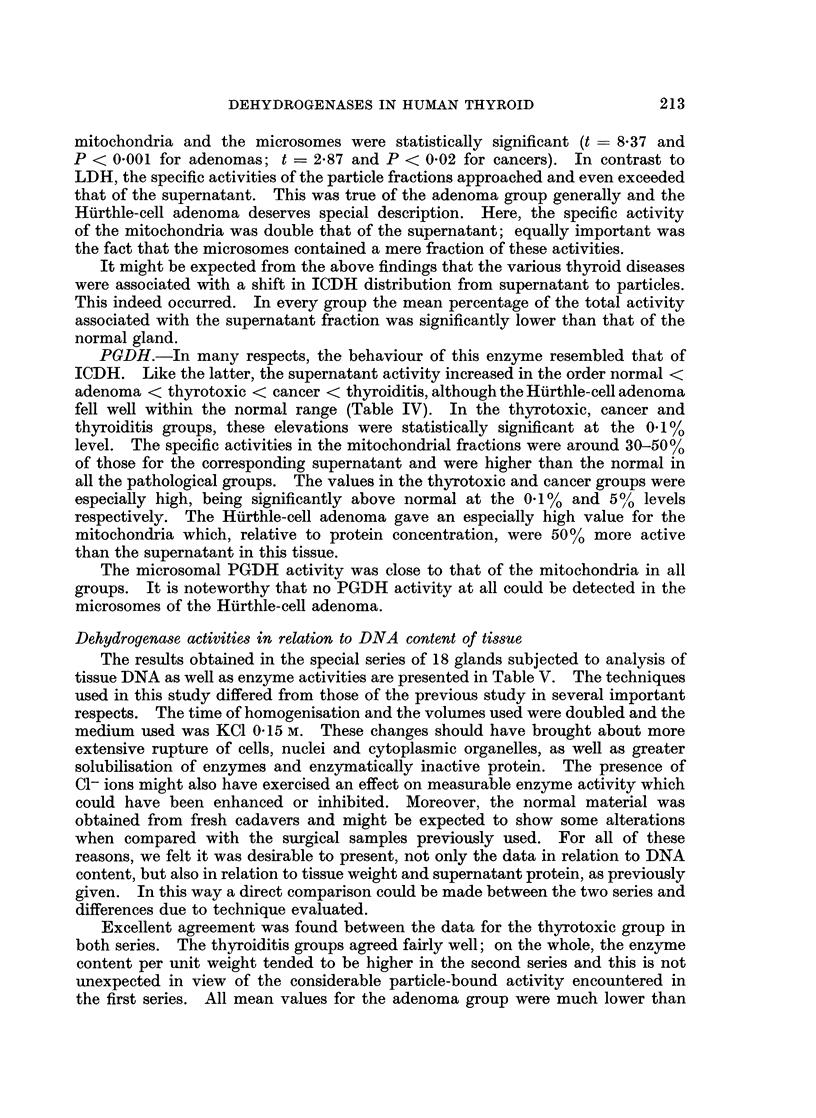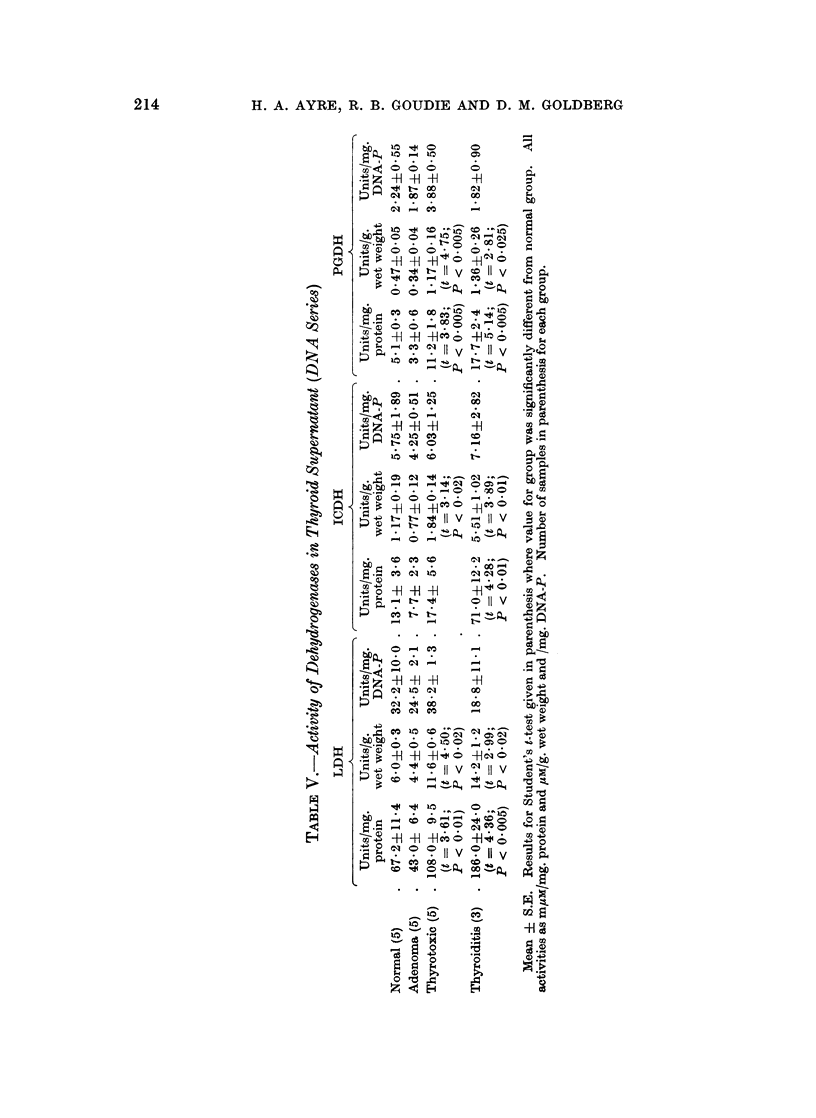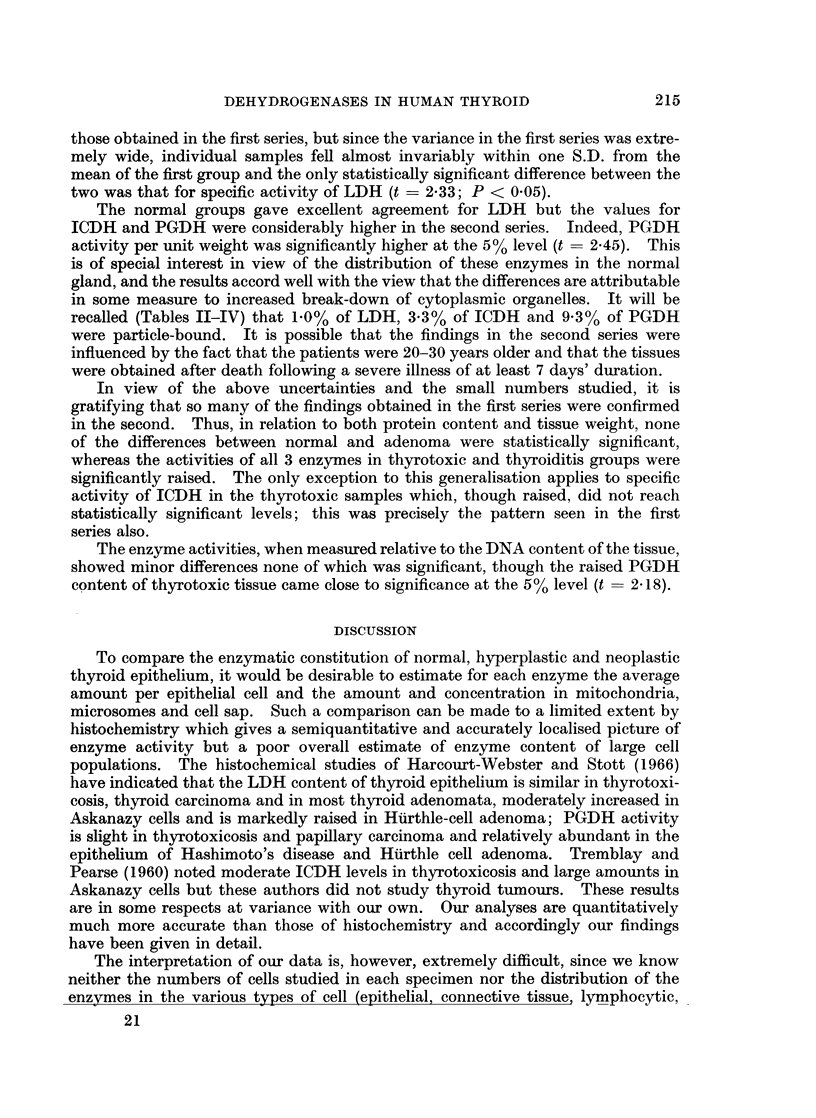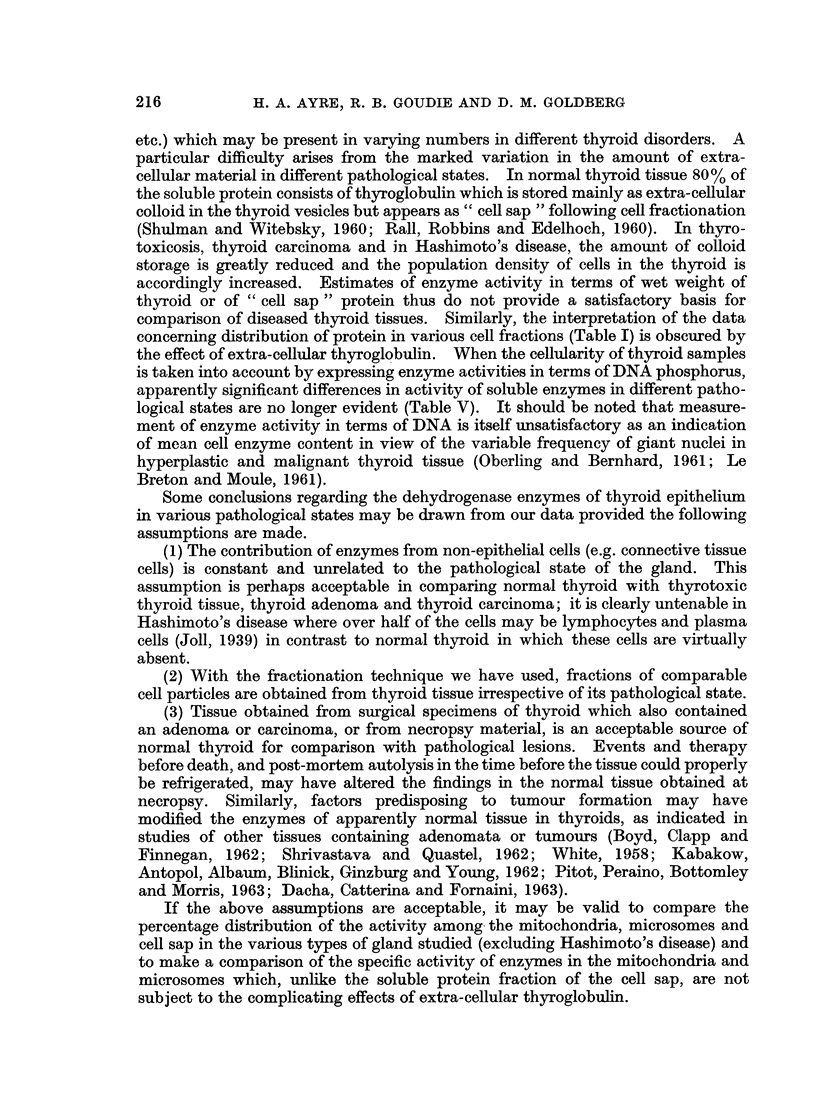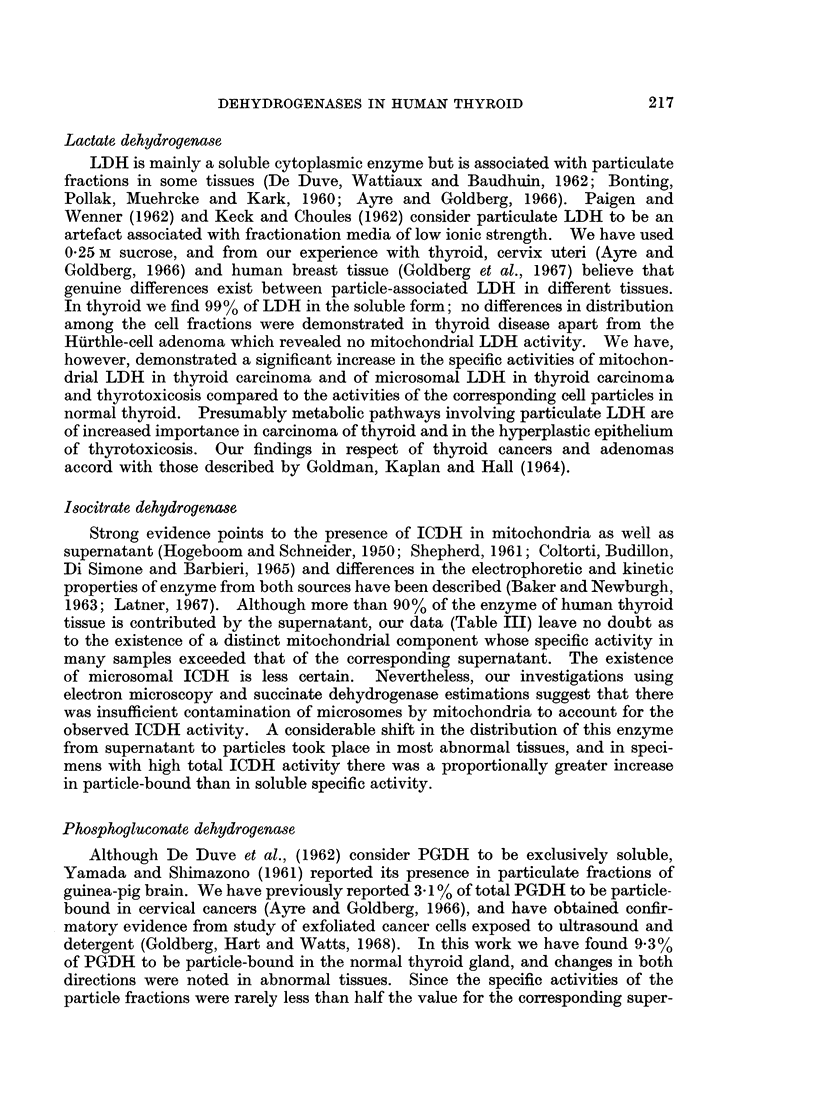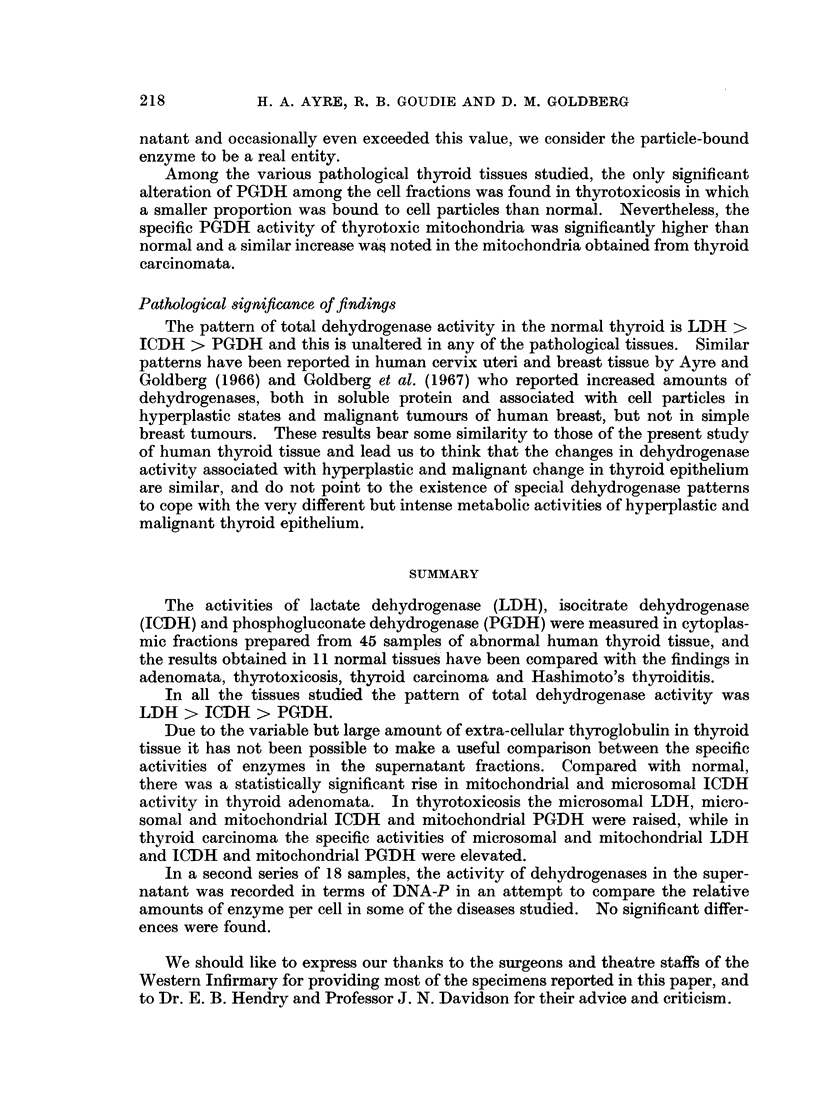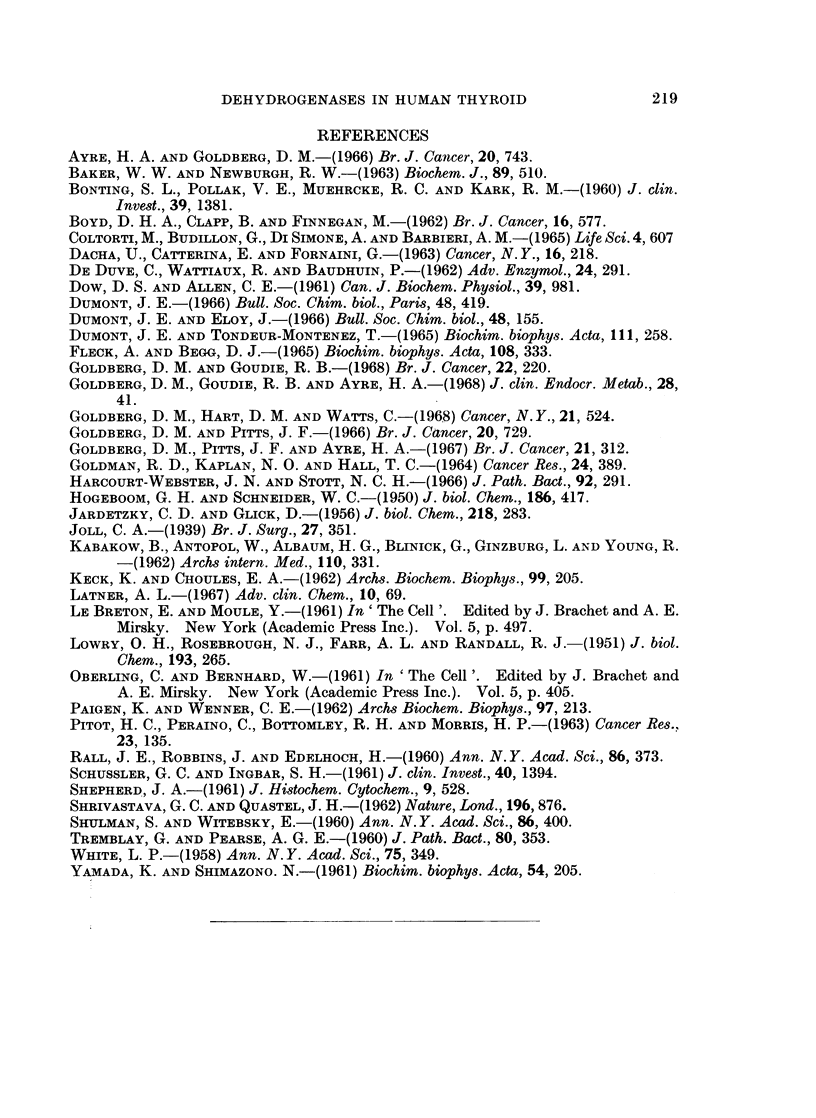# Effect of hyperplasia and neoplasia on the activity and distribution of some dehydrogenases in the human thyroid gland.

**DOI:** 10.1038/bjc.1968.28

**Published:** 1968-06

**Authors:** H. A. Ayre, R. B. Goudie, D. M. Goldberg


### EFFECT OF HYPERPLASIA AND NEOPLASIA ON THE ACTIVITY AND DISTRIBUTION OF SOME DEHYDROGENASES IN THE HUMAN THYROID GLAND

HEATHER A. AYRE, R. B. GOUDIE AND *D. M. GOLDBERG

*From the Department of Biochemistry, and the University Departments of Pathological Biochemistry and Pathology, Western Infirmary, Glasgow*

Received for publication January 24, 1968

PREVIOUS work in this Department has shown increased activities of certain dehydrogenases and nucleases in cancers of the human cervix uteri and breast (Goldberg and Pitts, 1966; Ayre and Goldberg, 1966; Goldberg, Pitts and Ayre, 1967). No differences were observed between simple breast tumours and normal tissue but many of the features found in the cancers were present in tissues showing diffuse epithelial hyperplasia. It seemed of considerable interest to determine whether these changes were generally characteristic of hyperplastic and neoplastic human epithelial tissues. The thyroid gland appeared a suitable organ in which to study the enzymological and cytochemical changes in which we were interested. Dow and Allen (1961), Schussler and Ingbar (1961), and Dumont and his associates (Dumont and Tondeur-Montenez, 1965; Dumont and Eloy, 1966; Dumont, 1966) have already presented evidence concerning the involvement of dehydrogenase enzymes and the energy pathways through which they operate in the metabolism of the thyroid gland in animals, although these studies have dealt mainly with the role of trophic hormones on various metabolic processes.

The present communication is concerned with our observations on the activity and distribution of dehydrogenases in hyperplastic and neoplastic diseases of the human thyroid gland and on the effect of these diseases upon the distribution of protein in the cytoplasm. Data on the nucleases are presented in a second report (Goldberg and Goudie, 1968).

### MATERIALS AND METHODS

The thyroid tissues used for study of the distribution of enzymes and cytoplasmic protein were fresh surgical specimens obtained within 30 minutes of removal from 5 categories of subjects.

*Normal.*—Eleven samples of histologically normal tissue were obtained from patients in whom the purpose of operation was removal of a thyroid adenoma or cancer.

*Thyrotoxic.*—Twenty specimens were obtained from patients who pre-operatively had received antithyroid drugs and iodine. These glands showed epithelial hyperplasia and areas of focal thyroiditis. While these changes were fairly uniform within a single gland, considerable histological variation from one gland to another was encountered.

* Present address: Department of Chemical Pathology, The Royal Hospital, West Street, Sheffield, 1.

*Adenoma.*—Eleven samples were examined. Nine were discrete solitary adenomata and 2 were from glands replaced by multiple adenomata which could not be separated. One sample consisted of a Hürthle-cell adenoma. The remaining 10 samples were extremely heterogeneous and displayed various degrees of cyst-formation, calcification, stromal degeneration, haemorrhage, necrosis and focal thyroiditis.

*Thyroiditis.*—Seven glands taken from patients with Hashimoto's thyroiditis were macroscopically homogeneous. On histological examination, they displayed the intense round-cell infiltration characteristic of this condition, with varying degrees of Askanazy cell change.

*Cancer.*—Seven samples were available. Six were primary tumours of which 4 were papillary adenocarcinomas and 2 were highly anaplastic undifferentiated tumours with many mitotic and aberrant cells. One of the adenocarcinomas was removed from a thyroid that was also the site of Hashimoto's thyroiditis. The seventh sample consisted of a metastasis in a cervical lymph node the substance of which was entirely replaced by thyroid adenocarcinoma. The primary tumour from this subject was too small for analysis.

All samples were washed with ice-cold distilled water until no further blood could be removed, whereupon they were blotted dry with absorbent paper, weighed and stored immediately at $-20°$ C. for up to 4 weeks. They were then minced in a pre-cooled domestic mincer, transferred quantitatively to a vessel containing 5 volumes of 0·25 M sucrose in crushed ice and homogenised for 3 minutes in a Model No. 7700 Blender (Measuring and Scientific Equipment Ltd.).

Nuclei and unbroken cells were sedimented from the homogenate by centrifugation at 500 ***g*** for 10 minutes. From the cytoplasm, three fractions were quantitatively prepared as previously described (Goldberg and Pitts, 1966):

Mitochondria—5,000 ***g*** for 20 minutes.
Microsomes—35,000 ***g*** for 60 minutes.

The unsedimented liquid surviving the last centrifugation represents the supernatant. These steps were carried out using the Superspeed 17 Refrigerated Centrifuge (Measuring and Scientific Equipment Ltd.). The particle preparations were made up to a known volume with ice-cold distilled water and dispersed at 20 kHz for 90 seconds under crushed ice using the Ultrasonic Disintegrator Model 60 W with titanium probe of $\frac{3}{8}$ inch end-diameter and 10 : 1 end-ratio (Measuring and Scientific Equipment Ltd.).

Preliminary experiments determined the conditions of particle disintegration as optimal for the enzymes under investigation. Electron microscopy on a typical tissue preparation showed that the nuclear pellet, which was discarded, was virtually free of mitochondria but contained strands of endoplasmic reticulum attached to the nuclear membrane. The second pellet comprised large and small mitochondria and was free of nuclear contamination but a small fraction of the material consisted of strands of endoplasmic reticulum with ribosomes attached, as well as occasional clusters of free ribosomes. The third pellet consisted predominantly of microsomal material and free ribosomes but about 10–15% of the various fields were composed of small mitochondria. Lysosomes were not commonly seen and were equally distributed between the second and third pellets. It was difficult to estimate their frequency but they never exceeded 4% of the particles in any field. Further studies involving estimation of ribonucleic acid

(Fleck and Begg, 1965) and succinate dehydrogenase (Jardetzky and Glick, 1956) in 3 thyroid tissues gave results in general agreement with the conclusions derived from electron microscopy and pointed to the mitochondrial being 90% and microsomal pellets being about 75–80% homogeneous for their respective particles. In these preparations, the supernatant after centrifugation at 35,000 *g* for 60 minutes was subjected to 105,000 *g* for 60 minutes in the Superspeed 50 Refrigerated Centrifuge (Measuring and Scientific Equipment Ltd.). Not more than 2% of the activity of any of the enzymes studied, and 3% of the protein, was sedimented under these conditions. We therefore concluded that the supernatant fractions were not contaminated with particulate elements to any significant extent.

In all fractions, protein was estimated by the Lowry method (Lowry, Rosebrough, Farr and Randall, 1951), and the activities of lactate dehydrogenase (LDH, EC 1.1.1.27), isocitrate dehydrogenase (ICDH, EC 1.1.1.42), and phosphogluconate dehydrogenase (PGDH, EC 1.1.1.44) were determined as previously described, units of activity in all cases being m$\mu$M substrate transformed/min. at 25° C. (Ayre and Goldberg, 1966). The activity of each fraction was expressed in relation to the protein content of the fraction, the wet weight of the tissue and the percentage of the total cytoplasmic activity attributable to that fraction. The protein content of each fraction was expressed in relation to the wet weight of the tissue and its percentage contribution to the total cytoplasmic protein.

A further series of glands was collected for the purpose of studying the relationship between enzyme content and cellularity. These were 5 fresh post-mortem glands from patients free of thyroid disease and fresh surgical samples from patients with adenomas (5 samples), thyrotoxicosis (5 samples) and Hashimoto's thyroiditis (3 samples). The clinical material and the methods used for estimation of deoxyribonucleic acid-phosphorus (DNA-P) have already been described (Goldberg, Goudie and Ayre, 1968). After mincing, the tissues were homogenised in 10 volumes ice-cold 0·15 M KCl using the Model No. 7700 Blendor for 5 minutes. Aliquots were taken for estimation of DNA-P and the remainder of the homogenate was centrifuged at 35,000 *g* for 60 minutes. The supernatant was collected for measurement of protein concentration and enzyme activities which were related to the wet weight and DNA-P content of the tissue and to the protein concentration of the supernatant.

### RESULTS

The protein estimations described below refer to soluble proteins derived from thyroid tissue excluding those contained in the nuclear fraction.

### *Distribution of protein in normal and diseased thyroid tissue*

In the normal thyroid gland, the supernatant contained more than 90% of the soluble protein (Table I). The protein content of all 3 fractions was slightly reduced in the thyrotoxic glands.

Although considerable variations occurred in the adenomas, a reduction in the supernatant protein content took place in most samples. A highly significant fall in the supernatant protein occurred in the cancers, and this was accompanied by a marked shift in distribution favouring the particle fractions.

More striking was the reduction in the supernatant protein content of the thyroiditis samples. As might be expected from the lack of colloid storage in this condition, the proportion of the cytoplasmic protein associated with the particles was significantly increased and the values relative to the tissue weight were also increased.

The single specimen of a Hürthle-cell adenoma is presented independently since some of the values obtained from this tissue lay beyond 3 S.D. from the mean of the remaining 10 adenomas. A marked reduction in the supernatant protein content of this sample was evident, the value being lower than that found in all but one of the 56 tissues examined. On the other hand, the mitochondria contained the highest amount of protein seen in this fraction in the entire series and accounted for more than one quarter of the soluble protein of the specimen.

*Enzyme activities and cytoplasmic distribution*

The activities of the dehydrogenases studied are presented as a function of protein concentration, wet weight of tissue, and percentage of total (non-nuclear) activity for each fraction in Tables II–IV. The mean and S.E. are given, and where these values differed significantly from the normal, the relevant statistical parameters are also shown. It should be noted that the S.E. is not recorded for the " percentage total activity " of the mitochondria and microsomes, as the values were usually extremely small; where a statistically significant shift in distribution occurred, this may readily be appreciated by reference to the supernatant fraction.

*LDH.*—The activity of this enzyme in the supernatant increased in the order normal < adenoma < thyrotoxic < cancer; this was true whether activity was related to protein concentration or wet weight of tissue (Table II). The same sequence was apparent in the mitochondria and microsomes. The specific activity of the supernatant was for most samples about 10-fold that of the particle fractions which did not average more than 2% of the total cytoplasmic activity in any one type of tissue. Differences between the mitochondria and microsomes were not large except in thyrotoxic tissue where the microsomes were almost twice as active as the mitochondria ($t = 3{\cdot}17$; $P < 0{\cdot}01$).

The values encountered in the thyroiditis samples approached those of the cancer group and were substantially higher than normal in every fraction. The Hürthle-cell adenoma had the highest supernatant specific activity of any tissue examined. For this reason, it is important to note that no LDH could be detected in the mitochondria, though the microsomes were four times as active as the mean value for the corresponding normal fraction.

*ICDH.*—As with the previous enzyme, ICDH activity was substantially higher than normal in every fraction of all the diseased tissues. In the supernatant, steadily increasing activity was found in the order normal < adenoma < thyrotoxic < cancer < thyroiditis < Hürthle-cell adenoma (Table III). Significantly raised values for ICDH were found in the mitochondria and microsomes of all the pathological groups. These increases were relatively much greater than those previously noted for LDH in these fractions, and they were proportionally much more dramatic than the extent of ICDH elevation recorded for the supernatants; tissues such as those in the adenoma and thyrotoxic group displayed ICDH levels in the particle fractions that were 3–6 times normal, whereas the supernatant content was merely doubled.

It should be noted that in every group the activity of the mitochondria was the greater. In the adenomas and in the cancers, these differences between the mitochondria and the microsomes were statistically significant ($t = 8{\cdot}37$ and $P < 0{\cdot}001$ for adenomas; $t = 2{\cdot}87$ and $P < 0{\cdot}02$ for cancers). In contrast to LDH, the specific activities of the particle fractions approached and even exceeded that of the supernatant. This was true of the adenoma group generally and the Hürthle-cell adenoma deserves special description. Here, the specific activity of the mitochondria was double that of the supernatant; equally important was the fact that the microsomes contained a mere fraction of these activities.

It might be expected from the above findings that the various thyroid diseases were associated with a shift in ICDH distribution from supernatant to particles. This indeed occurred. In every group the mean percentage of the total activity associated with the supernatant fraction was significantly lower than that of the normal gland.

*PGDH.*—In many respects, the behaviour of this enzyme resembled that of ICDH. Like the latter, the supernatant activity increased in the order normal < adenoma < thyrotoxic < cancer < thyroiditis, although the Hürthle-cell adenoma fell well within the normal range (Table IV). In the thyrotoxic, cancer and thyroiditis groups, these elevations were statistically significant at the 0·1% level. The specific activities in the mitochondrial fractions were around 30–50% of those for the corresponding supernatant and were higher than the normal in all the pathological groups. The values in the thyrotoxic and cancer groups were especially high, being significantly above normal at the 0·1% and 5% levels respectively. The Hürthle-cell adenoma gave an especially high value for the mitochondria which, relative to protein concentration, were 50% more active than the supernatant in this tissue.

The microsomal PGDH activity was close to that of the mitochondria in all groups. It is noteworthy that no PGDH activity at all could be detected in the microsomes of the Hürthle-cell adenoma.

*Dehydrogenase activities in relation to DNA content of tissue*

The results obtained in the special series of 18 glands subjected to analysis of tissue DNA as well as enzyme activities are presented in Table V. The techniques used in this study differed from those of the previous study in several important respects. The time of homogenisation and the volumes used were doubled and the medium used was KCl 0·15 M. These changes should have brought about more extensive rupture of cells, nuclei and cytoplasmic organelles, as well as greater solubilisation of enzymes and enzymatically inactive protein. The presence of $Cl^-$ ions might also have exercised an effect on measurable enzyme activity which could have been enhanced or inhibited. Moreover, the normal material was obtained from fresh cadavers and might be expected to show some alterations when compared with the surgical samples previously used. For all of these reasons, we felt it was desirable to present, not only the data in relation to DNA content, but also in relation to tissue weight and supernatant protein, as previously given. In this way a direct comparison could be made between the two series and differences due to technique evaluated.

Excellent agreement was found between the data for the thyrotoxic group in both series. The thyroiditis groups agreed fairly well; on the whole, the enzyme content per unit weight tended to be higher in the second series and this is not unexpected in view of the considerable particle-bound activity encountered in the first series. All mean values for the adenoma group were much lower than those obtained in the first series, but since the variance in the first series was extremely wide, individual samples fell almost invariably within one S.D. from the mean of the first group and the only statistically significant difference between the two was that for specific activity of LDH ($t = 2{\cdot}33$; $P < 0{\cdot}05$).

The normal groups gave excellent agreement for LDH but the values for ICDH and PGDH were considerably higher in the second series. Indeed, PGDH activity per unit weight was significantly higher at the 5% level ($t = 2{\cdot}45$). This is of special interest in view of the distribution of these enzymes in the normal gland, and the results accord well with the view that the differences are attributable in some measure to increased break-down of cytoplasmic organelles. It will be recalled (Tables II–IV) that 1·0% of LDH, 3·3% of ICDH and 9·3% of PGDH were particle-bound. It is possible that the findings in the second series were influenced by the fact that the patients were 20–30 years older and that the tissues were obtained after death following a severe illness of at least 7 days' duration.

In view of the above uncertainties and the small numbers studied, it is gratifying that so many of the findings obtained in the first series were confirmed in the second. Thus, in relation to both protein content and tissue weight, none of the differences between normal and adenoma were statistically significant, whereas the activities of all 3 enzymes in thyrotoxic and thyroiditis groups were significantly raised. The only exception to this generalisation applies to specific activity of ICDH in the thyrotoxic samples which, though raised, did not reach statistically significant levels; this was precisely the pattern seen in the first series also.

The enzyme activities, when measured relative to the DNA content of the tissue, showed minor differences none of which was significant, though the raised PGDH content of thyrotoxic tissue came close to significance at the 5% level ($t = 2{\cdot}18$).

### DISCUSSION

To compare the enzymatic constitution of normal, hyperplastic and neoplastic thyroid epithelium, it would be desirable to estimate for each enzyme the average amount per epithelial cell and the amount and concentration in mitochondria, microsomes and cell sap. Such a comparison can be made to a limited extent by histochemistry which gives a semiquantitative and accurately localised picture of enzyme activity but a poor overall estimate of enzyme content of large cell populations. The histochemical studies of Harcourt-Webster and Stott (1966) have indicated that the LDH content of thyroid epithelium is similar in thyrotoxicosis, thyroid carcinoma and in most thyroid adenomata, moderately increased in Askanazy cells and is markedly raised in Hürthle-cell adenoma; PGDH activity is slight in thyrotoxicosis and papillary carcinoma and relatively abundant in the epithelium of Hashimoto's disease and Hürthle cell adenoma. Tremblay and Pearse (1960) noted moderate ICDH levels in thyrotoxicosis and large amounts in Askanazy cells but these authors did not study thyroid tumours. These results are in some respects at variance with our own. Our analyses are quantitatively much more accurate than those of histochemistry and accordingly our findings have been given in detail.

The interpretation of our data is, however, extremely difficult, since we know neither the numbers of cells studied in each specimen nor the distribution of the enzymes in the various types of cell (epithelial, connective tissue, lymphocytic, etc.) which may be present in varying numbers in different thyroid disorders. A particular difficulty arises from the marked variation in the amount of extra-cellular material in different pathological states. In normal thyroid tissue 80% of the soluble protein consists of thyroglobulin which is stored mainly as extra-cellular colloid in the thyroid vesicles but appears as " cell sap " following cell fractionation (Shulman and Witebsky, 1960; Rall, Robbins and Edelhoch, 1960). In thyrotoxicosis, thyroid carcinoma and in Hashimoto's disease, the amount of colloid storage is greatly reduced and the population density of cells in the thyroid is accordingly increased. Estimates of enzyme activity in terms of wet weight of thyroid or of " cell sap " protein thus do not provide a satisfactory basis for comparison of diseased thyroid tissues. Similarly, the interpretation of the data concerning distribution of protein in various cell fractions (Table I) is obscured by the effect of extra-cellular thyroglobulin. When the cellularity of thyroid samples is taken into account by expressing enzyme activities in terms of DNA phosphorus, apparently significant differences in activity of soluble enzymes in different pathological states are no longer evident (Table V). It should be noted that measurement of enzyme activity in terms of DNA is itself unsatisfactory as an indication of mean cell enzyme content in view of the variable frequency of giant nuclei in hyperplastic and malignant thyroid tissue (Oberling and Bernhard, 1961; Le Breton and Moule, 1961).

Some conclusions regarding the dehydrogenase enzymes of thyroid epithelium in various pathological states may be drawn from our data provided the following assumptions are made.

(1) The contribution of enzymes from non-epithelial cells (e.g. connective tissue cells) is constant and unrelated to the pathological state of the gland. This assumption is perhaps acceptable in comparing normal thyroid with thyrotoxic thyroid tissue, thyroid adenoma and thyroid carcinoma; it is clearly untenable in Hashimoto's disease where over half of the cells may be lymphocytes and plasma cells (Joll, 1939) in contrast to normal thyroid in which these cells are virtually absent.

(2) With the fractionation technique we have used, fractions of comparable cell particles are obtained from thyroid tissue irrespective of its pathological state.

(3) Tissue obtained from surgical specimens of thyroid which also contained an adenoma or carcinoma, or from necropsy material, is an acceptable source of normal thyroid for comparison with pathological lesions. Events and therapy before death, and post-mortem autolysis in the time before the tissue could properly be refrigerated, may have altered the findings in the normal tissue obtained at necropsy. Similarly, factors predisposing to tumour formation may have modified the enzymes of apparently normal tissue in thyroids, as indicated in studies of other tissues containing adenomata or tumours (Boyd, Clapp and Finnegan, 1962; Shrivastava and Quastel, 1962; White, 1958; Kabakow, Antopol, Albaum, Blinick, Ginzburg and Young, 1962; Pitot, Peraino, Bottomley and Morris, 1963; Dacha, Catterina and Fornaini, 1963).

If the above assumptions are acceptable, it may be valid to compare the percentage distribution of the activity among the mitochondria, microsomes and cell sap in the various types of gland studied (excluding Hashimoto's disease) and to make a comparison of the specific activity of enzymes in the mitochondria and microsomes which, unlike the soluble protein fraction of the cell sap, are not subject to the complicating effects of extra-cellular thyroglobulin.

*Lactate dehydrogenase*

LDH is mainly a soluble cytoplasmic enzyme but is associated with particulate fractions in some tissues (De Duve, Wattiaux and Baudhuin, 1962; Bonting, Pollak, Muehrcke and Kark, 1960; Ayre and Goldberg, 1966). Paigen and Wenner (1962) and Keck and Choules (1962) consider particulate LDH to be an artefact associated with fractionation media of low ionic strength. We have used 0·25 M sucrose, and from our experience with thyroid, cervix uteri (Ayre and Goldberg, 1966) and human breast tissue (Goldberg *et al.*, 1967) believe that genuine differences exist between particle-associated LDH in different tissues. In thyroid we find 99% of LDH in the soluble form; no differences in distribution among the cell fractions were demonstrated in thyroid disease apart from the Hürthle-cell adenoma which revealed no mitochondrial LDH activity. We have, however, demonstrated a significant increase in the specific activities of mitochondrial LDH in thyroid carcinoma and of microsomal LDH in thyroid carcinoma and thyrotoxicosis compared to the activities of the corresponding cell particles in normal thyroid. Presumably metabolic pathways involving particulate LDH are of increased importance in carcinoma of thyroid and in the hyperplastic epithelium of thyrotoxicosis. Our findings in respect of thyroid cancers and adenomas accord with those described by Goldman, Kaplan and Hall (1964).

*Isocitrate dehydrogenase*

Strong evidence points to the presence of ICDH in mitochondria as well as supernatant (Hogeboom and Schneider, 1950; Shepherd, 1961; Coltorti, Budillon, Di Simone and Barbieri, 1965) and differences in the electrophoretic and kinetic properties of enzyme from both sources have been described (Baker and Newburgh, 1963; Latner, 1967). Although more than 90% of the enzyme of human thyroid tissue is contributed by the supernatant, our data (Table III) leave no doubt as to the existence of a distinct mitochondrial component whose specific activity in many samples exceeded that of the corresponding supernatant. The existence of microsomal ICDH is less certain. Nevertheless, our investigations using electron microscopy and succinate dehydrogenase estimations suggest that there was insufficient contamination of microsomes by mitochondria to account for the observed ICDH activity. A considerable shift in the distribution of this enzyme from supernatant to particles took place in most abnormal tissues, and in specimens with high total ICDH activity there was a proportionally greater increase in particle-bound than in soluble specific activity.

*Phosphogluconate dehydrogenase*

Although De Duve *et al.*, (1962) consider PGDH to be exclusively soluble, Yamada and Shimazono (1961) reported its presence in particulate fractions of guinea-pig brain. We have previously reported 3·1% of total PGDH to be particle-bound in cervical cancers (Ayre and Goldberg, 1966), and have obtained confirmatory evidence from study of exfoliated cancer cells exposed to ultrasound and detergent (Goldberg, Hart and Watts, 1968). In this work we have found 9·3% of PGDH to be particle-bound in the normal thyroid gland, and changes in both directions were noted in abnormal tissues. Since the specific activities of the particle fractions were rarely less than half the value for the corresponding supernatant and occasionally even exceeded this value, we consider the particle-bound enzyme to be a real entity.

Among the various pathological thyroid tissues studied, the only significant alteration of PGDH among the cell fractions was found in thyrotoxicosis in which a smaller proportion was bound to cell particles than normal. Nevertheless, the specific PGDH activity of thyrotoxic mitochondria was significantly higher than normal and a similar increase was noted in the mitochondria obtained from thyroid carcinomata.

*Pathological significance of findings*

The pattern of total dehydrogenase activity in the normal thyroid is LDH > ICDH > PGDH and this is unaltered in any of the pathological tissues. Similar patterns have been reported in human cervix uteri and breast tissue by Ayre and Goldberg (1966) and Goldberg *et al.* (1967) who reported increased amounts of dehydrogenases, both in soluble protein and associated with cell particles in hyperplastic states and malignant tumours of human breast, but not in simple breast tumours. These results bear some similarity to those of the present study of human thyroid tissue and lead us to think that the changes in dehydrogenase activity associated with hyperplastic and malignant change in thyroid epithelium are similar, and do not point to the existence of special dehydrogenase patterns to cope with the very different but intense metabolic activities of hyperplastic and malignant thyroid epithelium.

### SUMMARY

The activities of lactate dehydrogenase (LDH), isocitrate dehydrogenase (ICDH) and phosphogluconate dehydrogenase (PGDH) were measured in cytoplasmic fractions prepared from 45 samples of abnormal human thyroid tissue, and the results obtained in 11 normal tissues have been compared with the findings in adenomata, thyrotoxicosis, thyroid carcinoma and Hashimoto's thyroiditis.

In all the tissues studied the pattern of total dehydrogenase activity was LDH > ICDH > PGDH.

Due to the variable but large amount of extra-cellular thyroglobulin in thyroid tissue it has not been possible to make a useful comparison between the specific activities of enzymes in the supernatant fractions. Compared with normal, there was a statistically significant rise in mitochondrial and microsomal ICDH activity in thyroid adenomata. In thyrotoxicosis the microsomal LDH, microsomal and mitochondrial ICDH and mitochondrial PGDH were raised, while in thyroid carcinoma the specific activities of microsomal and mitochondrial LDH and ICDH and mitochondrial PGDH were elevated.

In a second series of 18 samples, the activity of dehydrogenases in the supernatant was recorded in terms of DNA-*P* in an attempt to compare the relative amounts of enzyme per cell in some of the diseases studied. No significant differences were found.

We should like to express our thanks to the surgeons and theatre staffs of the Western Infirmary for providing most of the specimens reported in this paper, and to Dr. E. B. Hendry and Professor J. N. Davidson for their advice and criticism.

### REFERENCES

AYRE, H. A. AND GOLDBERG, D. M.—(1966) *Br. J. Cancer*, **20**, 743.
BAKER, W. W. AND NEWBURGH, R. W.—(1963) *Biochem. J.*, **89**, 510.
BONTING, S. L., POLLAK, V. E., MUEHRCKE, R. C. AND KARK, R. M.—(1960) *J. clin. Invest.*, **39**, 1381.
BOYD, D. H. A., CLAPP, B. AND FINNEGAN, M.—(1962) *Br. J. Cancer*, **16**, 577.
COLTORTI, M., BUDILLON, G., DI SIMONE, A. AND BARBIERI, A. M.—(1965) *Life Sci.* **4**, 607
DACHA, U., CATTERINA, E. AND FORNAINI, G.—(1963) *Cancer, N.Y.*, **16**, 218.
DE DUVE, C., WATTIAUX, R. AND BAUDHUIN, P.—(1962) *Adv. Enzymol.*, **24**, 291.
DOW, D. S. AND ALLEN, C. E.—(1961) *Can. J. Biochem. Physiol.*, **39**, 981.
DUMONT, J. E.—(1966) *Bull. Soc. Chim. biol., Paris*, 48, 419.
DUMONT, J. E. AND ELOY, J.—(1966) *Bull. Soc. Chim. biol.*, **48**, 155.
DUMONT, J. E. AND TONDEUR-MONTENEZ, T.—(1965) *Biochim. biophys. Acta*, **111**, 258.
FLECK, A. AND BEGG, D. J.—(1965) *Biochim. biophys. Acta*, **108**, 333.
GOLDBERG, D. M. AND GOUDIE, R. B.—(1968) *Br. J. Cancer*, **22**, 220.
GOLDBERG, D. M., GOUDIE, R. B. AND AYRE, H. A.—(1968) *J. clin. Endocr. Metab.*, **28**, 41.
GOLDBERG, D. M., HART, D. M. AND WATTS, C.—(1968) *Cancer, N.Y.*, **21**, 524.
GOLDBERG, D. M. AND PITTS, J. F.—(1966) *Br. J. Cancer*, **20**, 729.
GOLDBERG, D. M., PITTS, J. F. AND AYRE, H. A.—(1967) *Br. J. Cancer*, **21**, 312.
GOLDMAN, R. D., KAPLAN, N. O. AND HALL, T. C.—(1964) *Cancer Res.*, **24**, 389.
HARCOURT-WEBSTER, J. N. AND STOTT, N. C. H.—(1966) *J. Path. Bact.*, **92**, 291.
HOGEBOOM, G. H. AND SCHNEIDER, W. C.—(1950) *J. biol. Chem.*, **186**, 417.
JARDETZKY, C. D. AND GLICK, D.—(1956) *J. biol. Chem.*, **218**, 283.
JOLL, C. A.—(1939) *Br. J. Surg.*, **27**, 351.
KABAKOW, B., ANTOPOL, W., ALBAUM, H. G., BLINICK, G., GINZBURG, L. AND YOUNG, R. —(1962) *Archs intern. Med.*, **110**, 331.
KECK, K. AND CHOULES, E. A.—(1962) *Archs. Biochem. Biophys.*, **99**, 205.
LATNER, A. L.—(1967) *Adv. clin. Chem.*, **10**, 69.
LE BRETON, E. AND MOULE, Y.—(1961) *In* ' The Cell '. Edited by J. Brachet and A. E. Mirsky. New York (Academic Press Inc.). Vol. 5, p. 497.
LOWRY, O. H., ROSEBROUGH, N. J., FARR, A. L. AND RANDALL, R. J.—(1951) *J. biol. Chem.*, **193**, 265.
OBERLING, C. AND BERNHARD, W.—(1961) *In* ' The Cell '. Edited by J. Brachet and A. E. Mirsky. New York (Academic Press Inc.). Vol. 5, p. 405.
PAIGEN, K. AND WENNER, C. E.—(1962) *Archs Biochem. Biophys.*, **97**, 213.
PITOT, H. C., PERAINO, C., BOTTOMLEY, R. H. AND MORRIS, H. P.—(1963) *Cancer Res.*, **23**, 135.
RALL, J. E., ROBBINS, J. AND EDELHOCH, H.—(1960) *Ann. N.Y. Acad. Sci.*, **86**, 373.
SCHUSSLER, G. C. AND INGBAR, S. H.—(1961) *J. clin. Invest.*, **40**, 1394.
SHEPHERD, J. A.—(1961) *J. Histochem. Cytochem.*, **9**, 528.
SHRIVASTAVA, G. C. AND QUASTEL, J. H.—(1962) *Nature, Lond.*, **196**, 876.
SHULMAN, S. AND WITEBSKY, E.—(1960) *Ann. N.Y. Acad. Sci.*, **86**, 400.
TREMBLAY, G. AND PEARSE, A. G. E.—(1960) *J. Path. Bact.*, **80**, 353.
WHITE, L. P.—(1958) *Ann. N.Y. Acad. Sci.*, **75**, 349.
YAMADA, K. AND SHIMAZONO. N.—(1961) *Biochim. biophys. Acta*, **54**, 205.